# Imbalanced Protein Expression Patterns of Anabolic, Catabolic, Anti-Catabolic and Inflammatory Cytokines in Degenerative Cervical Disc Cells: New Indications for Gene Therapeutic Treatments of Cervical Disc Diseases

**DOI:** 10.1371/journal.pone.0096870

**Published:** 2014-05-07

**Authors:** Demissew S. Mern, Anja Beierfuß, Johann Fontana, Claudius Thomé, Aldemar A. Hegewald

**Affiliations:** 1 Department of Neurosurgery, Innsbruck Medical University, Innsbruck, Tirol, Austria; 2 Department of Neurosurgery, University Medical Center Mannheim, Heidelberg University, Mannheim, Baden-Württemberg, Germany; National Centre for Scientific Research, 'Demokritos', Greece

## Abstract

Degenerative disc disease (DDD) of the cervical spine is common after middle age and can cause loss of disc height with painful nerve impingement, bone and joint inflammation. Despite the clinical importance of these problems, in current publications the pathology of cervical disc degeneration has been studied merely from a morphologic view point using magnetic resonance imaging (MRI), without addressing the issue of biological treatment approaches. So far a wide range of endogenously expressed bioactive factors in degenerative cervical disc cells has not yet been investigated, despite its importance for gene therapeutic approaches. Although degenerative lumbar disc cells have been targeted by different biological treatment approaches, the quantities of disc cells and the concentrations of gene therapeutic factors used in animal models differ extremely. These indicate lack of experimentally acquired data regarding disc cell proliferation and levels of target proteins. Therefore, we analysed proliferation and endogenous expression levels of anabolic, catabolic, ant-catabolic, inflammatory cytokines and matrix proteins of degenerative cervical disc cells in three-dimensional cultures. Preoperative MRI grading of cervical discs was used, then grade III and IV nucleus pulposus (NP) tissues were isolated from 15 patients, operated due to cervical disc herniation. NP cells were cultured for four weeks with low-glucose in collagen I scaffold. Their proliferation rates were analysed using 3-(4, 5-dimethylthiazolyl-2)-2,5-diphenyltetrazolium bromide. Their protein expression levels of 28 therapeutic targets were analysed using enzyme-linked immunosorbent assay. During progressive grades of degeneration NP cell proliferation rates were similar. Significantly decreased aggrecan and collagen II expressions (P<0.0001) were accompanied by accumulations of selective catabolic and inflammatory cytokines (disintegrin and metalloproteinase with thrombospondin motifs 4 and 5, matrix metalloproteinase 3, interleukin-1β, interleukin-1 receptor) combined with low expression of anti-catabolic factor (metalloproteinase inhibitor 3) (P<0.0001). This study might contribute to inhibit inflammatory catabolism of cervical discs.

## Introduction

After middle age many people experience pain symptoms of cervical disc degeneration. Pain symptoms may get worse over time, which are accompanied by loss of disc height, painful nerve impingement, bone and joint inflammation [1]–[6]. The degenerative process can cause radiating pain and loss of mobility that have a major impact on the quality of life. Previous publications have analysed the pathology of cervical disc degeneration only from a morphologic view point using magnetic resonance imaging (MRI), which does not address the issue of biological treatment approaches. Until now the expression patterns of extracellular matrix (ECM) associated proteins in cervical nucleus pulposus cells are not published. Our current work is the first investigation concerning the endogenous expression patterns of ECM-associated proteins in degenerative cervical disc cells. Considerable anatomical differences between cervical and lumbar discs have been previously presented [7]–[8]. Furthermore, Mechanical properties in cervical discs have shown specific features and demonstrated some differences from lumbar discs [9]–[10]. The anatomical and mechanical differences might lead to functional modifications in cervical disc cells. These reasons suggest that biomolecular results from lumbar disc cells should not be directly projected onto cervical disc cells without any similar investigations. Numerous investigations have been made in lumbar discs to understand how bioactive factors combine to promote painful disc degeneration [11]–[12]. However, previous publications have not yet displayed the biomolecular differences or similarities between lumbar and cervical disc. Thus, the data of the current study address for the first time the biomolecular issue of cervical disc degeneration and might contribute valuably to gene therapeutic approaches of painful intervertebral disc degeneration.

Degenerative lumbar intervertebral discs (IVDs) have been targeted by different biological treatment approaches. Nucleus pulposus (NP) cells have been shown to play a central role in the maintenance of lumbar IVDs by organizing the expression of anabolic, catabolic, anti-catabolic and inflammatory cytokines that affect the synthesis and degradation of the IVD matrix. IVD degeneration is shown to be associated with imbalances of these factors combined with the declined cell density in adult IVDs [11]–[23]. Nevertheless, the amounts of lumbar NP cells and the concentrations of gene therapeutic factors used for regeneration of IVD tissues in animal models differ extremely [11]–[16]. These exhibit lack of experimentally acquired data regarding proliferation rates of NP cells and their endogenous expression levels of therapeutic target proteins. Recently we have reported about proliferation rates and imbalances of anabolic and catabolic factors regarding adult lumbar NP cells, and suggested potentially useful gene therapeutic targets [24].

So far a wide range of endogenously expressed bioactive factors, which are vital for designing objective gene therapeutic approaches, has not yet been investigated in degenerative cervical disc cells. Thus, we analysed proliferation rates of degenerative cervical NP cells and their endogenous expression levels of therapeutic target proteins in a three-dimensional collagen I scaffold. Since spinal disc herniation in adults predominantly occurs in discs of degeneration grade III and IV, we analysed cervical NP cells from those patients of disc degeneration grade III and IV, operated due to cervical disc herniation. Patient inclusion criteria for surgery were radiographically determined cervical disc herniation with nerve root compression on MRI, which correlated to primary symptoms that remained unresponsive to non-operative treatment for six weeks or demonstrated progressive neurological deterioration in the face of conservative treatment.

Progressive grade of cervical disc degeneration is significantly associated with accumulation or low expression levels of selective bioactive factors, which might cause unfavourable phenotypic alternations that might impair IVDs regeneration. The results of this study might contribute to design objective gene therapeutic treatment approaches and help to inhibit the inflammatory catabolism of intervertebral disc tissue.

## Materials and Methods

### Cervical IVD Specimens

Cervical nucleus pulposus tissues were acquired from patients with informed consents. Patients were operated due to cervical disc herniation. Participants provided their written informed consent to participate in this study. Experimental studies of human cervical IVD specimens were approved by the local research ethics committee (Heidelberg University, University Medical Center Mannheim: project 2009-217N-MA). The Miyazaki MRI scoring system [25] was used to determine the degeneration grades of cervical IVDs. 15 Patients (15 cervical IVDs of degeneration grade III and IV) with a mean age of 55 years (range 35–75 years) were involved (table 1). For isolation of NP cells, residual NP tissues from cervical disc space were recruited and brought immediately to the lab in sterile phosphate buffered saline solution (PBS) (Sigma-Aldrich).

**Table 1:** Details of cervical disc specimens.

Tissue No.	Disc Level	DDG	Age/Gender
1	C4/5	III	35/M
2	C5/6	III	43/F
3	C4/5	III	48/M
4	C5/6	III	52/F
5	C5/6	III	53/F
6	C5/6	III	54/F
7	C5/6	III	55/M
8	C6/7	IV	44/F
9	C5/6	IV	44/F
10	C5/6	IV	56/M
11	C6/7	IV	64/M
12	C4/5	IV	66/F
13	C5/6	IV	66/F
14	C5/6	IV	67/M
15	C5/6	IV	75/F

Residual NP tissues of degeneration grade III and IV were isolated from the disc space during surgical procedure conducted on 15 patients with cervical disc herniation. DDG: disc degeneration grade.

### Isolation and Monolayer Culture of Cervical NP Cell

Isolation and monolayer expansion of cervical NP cells were performed as described in our previous publication [24]. Briefly, NP specimens were washed in PBS and carefully separated from AF tissues. NP tissues were then minced into small fragments of approximately 2 mm^3^ and sequentially digested with pronase, collagenase II and hyaluronidase. After filtration of the samples through nylon mesh filters (75 gm), supernatants were centrifuged and pellets were suspended in 10 ml Dulbecco's Modified Eagle's Medium (DMEM) containing 1% v/v penicillin/streptomycin, 1% w/v glucose and 10% v/v FCS. By changing the culture medium every two day, NP cells were cultured for 2 weeks in 75 cm^2^ tissue culture flask. Monolayer expanded cervical NP cells were then cryopreserved at −196°C in culture medium containing 30% v/v FCS and 15% v/v dimethyl sulfoxide (DMSO).

For control two-dimensional (2D) culture of cervical NP cells in tissue culture dishes (100×20 mm, Sigma-Aldrich), 4×10^5^ NP cells were seeded in 10 ml DMEM containing 1% v/v penicillin/streptomycin, 1% w/v glucose and 10% v/v FCS. Cells were cultured for four weeks (37°C, 5% CO2) by changing the culture medium every two days.

### Three-dimensional Culture of Cervical NP Cells

The three-dimensional (3D) culture of cervical NP cells in collagen I based cell carrier (CCC) was carried out as previously described [24]. For control 3D culture of cervical NP cells in agarose gels, 6-well plates were coated with a thin layer of 1% agar (Sigma-Aldrich). 2% agarose of high electroendosmosis (Sigma-Aldrich) was autoclaved and equilibrated to 37°C. The agarose was mixed with equal volume of DMEM containing 1% v/v penicillin/streptomycin, 1% w/v glucose and 10% v/v FCS. Then the solution was mixed with one volume part of cell suspension to yield a final density of 4×10^5^ cells per milliliter and 1 ml was added to the pre-coated 6-well plates. Following gelation (4°C, 10 min) the embedded cells were overlaid with culture medium. Cells were cultured for four weeks (37°C, 5% CO2) by changing the culture medium every two days. The NP cells were then processed for control quantification of collagen I expression.

### Isolation of 3D Cultured Cervical NP Cells

Isolation of three-dimensional cultured cervical NP cells from collagen I scaffold was performed as formerly described [24]. To isolate three-dimensional cultured cervical NP cells from control agarose gels, the AgarACE Agarose-Digesting Enzyme (Promega) was used. Each 200 mg of agarose gel slice was transferred to a 1.5 ml micro-centrifuge tube and melted for 10 min at 65°C. After whirling for 2 seconds, the tube was transferred to a 42°C heating block and the gel was digested for 15 min with AgarACE Agarose-Digesting Enzyme (2U). After digestion of the gels, samples were filtered through nylon mesh filter (75 gm), supernatants were centrifuged for 2 min (1000×g) and cell pellets were washed twice in PBS for 2 min (1000×g). The NP cells were then processed for control quantification of collagen I expression.

### Proliferation Assay of Cervical NP Cells

As previously described [24] the MTT assay was applied to determine the proliferation of cervical NP cells. Briefly, NP cells were suspended in 0.5 ml culture medium and 100 µl duplicates of cell suspension were plated into flat-bottomed 96 well plates in addition to duplicate of blank control wells of medium alone. After 24 h of incubation, MTT reagent (10 µl) was added to each well. Following 3 h of incubation, 100 µl SDS-HCl solution was added for additional incubation of 4 h. The average absorbance value (570 nm) of the blank duplicate readings was subtracted from the average values of the sample duplicate readings and cell concentration was calculated from the standard curve. Cervical NP cell proliferation data represent the mean of at least three individual experiments.

### Isolation and Quantification of Target Proteins from Cervical NP Cells

For the isolation and quantification of target proteins from cervical NP cells, 4×10^5^ cells were cultured for four weeks in collagen I scaffold, TC dish or agarose gel. NP cell pellets were harvested and pellets were washed twice for 5 min in cold PBS (2500×g). Proteins were isolated by using the radio-immunoprecipitation assay (RIPA) buffer as formerly described [24]. Protein concentrations in samples were determined according to the instruction manual (Pierce Micro BCA Protein Assay Protocol) (Thermo Scientific).

### Enzyme-linked Immunosorbant Assay of Target Proteins

To determine the concentration of the target proteins in degenerative cervical NP cells, the enzyme-linked immunosorbant assay (ELISA) was applied on 100 µg of total protein extracts from each sample for each experiment as described before [24]. Briefly, the endogenous protein expression levels of following 28 target genes were analyzed. The catabolic factors: matrix metalloproteinase (MMP-1, - 2, -3, -7, -8, -9, -10 and -13) and a disintegrin and metalloproteinase with thrombospondin motifs (ADAMTS-4 and -5); anti-catabolic factors: metalloproteinase inhibitor (TIMP-1, -2, -3 and -4); the inflammatory cytokines: interleukin-1β (IL-1β), interleukin-1 receptor (IL-1 R1), tumor necrosis factor-α (TNF-α), tumor necrosis factor receptor R1 (TNF-R1); anabolic factors: bone morphogenetic proteins (BMP-2, -4, -6 and -7), insulin-like growth factor 1 (IGF-1), transforming growth factor betas (TGF-β1 and 3); and matrix proteins: aggrecan, collagen I and II. Cervical NP cell protein expression data represent the mean of at least three individual experiments.

### Statistical Data Analysis

Landis and Koch [25]–[27] based interpretations with κ statistics and agreement percentage among two observers (interobserver reliability) were applied to estimate the reliability of the MRI evaluations. Frequency of disagreement was calculated for each degeneration grade. The software IBM SPSS Statistics 20, Armonk New York USA was applied for statistical analysis. 1-way ANOVA and pairwise comparisons were used to analyze cell proliferation rates and protein expression levels as a function of degeneration grade, age and gender. Significance in all cases was set at P<0.05.

## Results

### Interobserver Reliability of MRI-grading

The interobserver agreement was excellent (κ = 0.885) and the calculated frequency of agreement was 92.31%.

### Degenerative Cervical NP Cell Proliferation Rates

**Figure 1:**
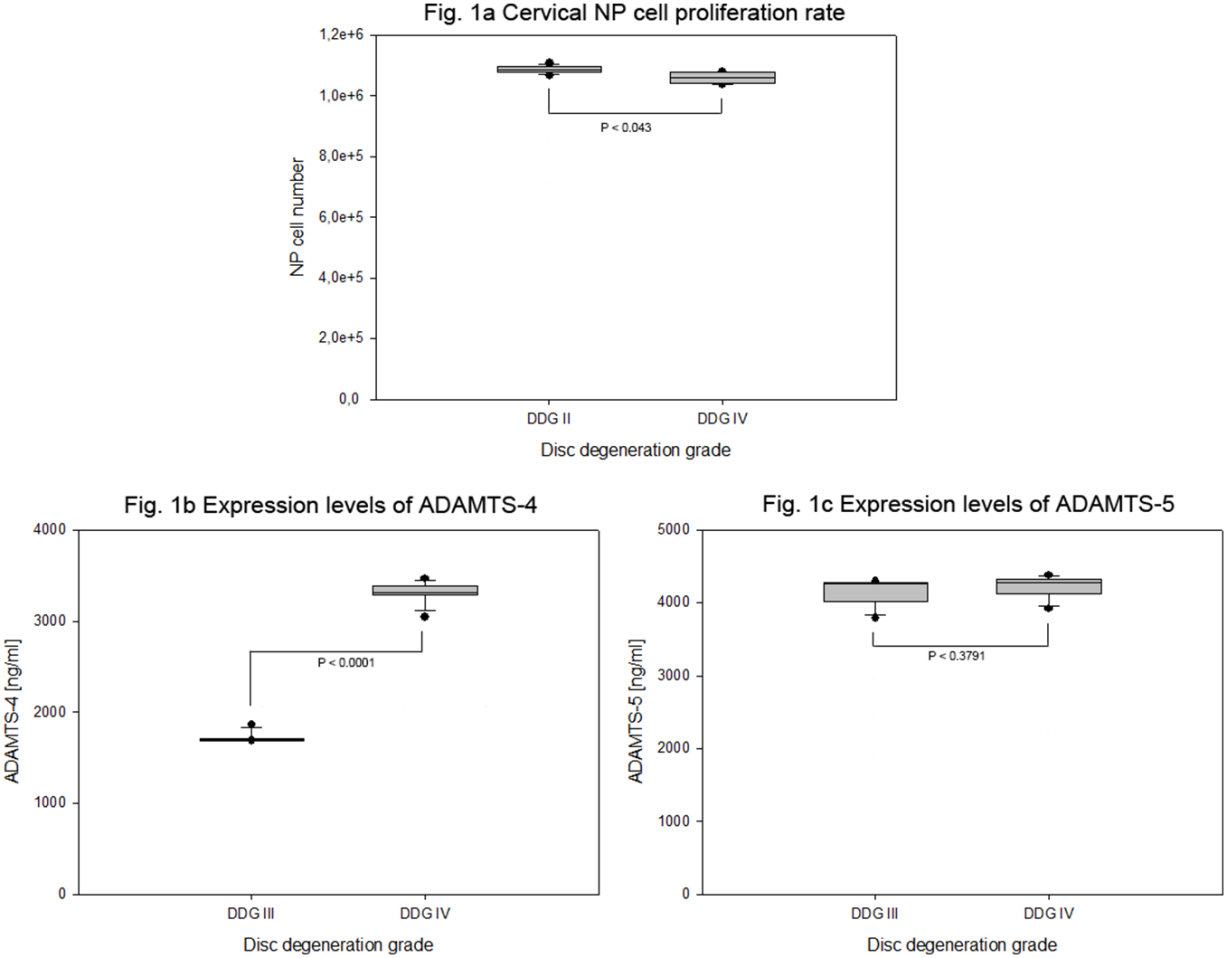
Proliferation rates of degenerative cervical NP cells and their endogenous protein expression levels of ADAMTS-4 and ADAMTS-5. Cervical NP cells were isolated from 15 cervical disc specimens of degenerative grade III and IV. 4×10^5^ cells from each specimen were grown for four weeks in collagen I scaffold. NP cell proliferation rates (MTT assay) and endogenous protein expression levels (ELISA) of ADAMTS-4 and ADAMTS-5 from 100 µg total protein extracts were analyzed on the basis of disc degeneration grade (DDG). Box plots with whiskers min to max show NP cell proliferation rates (Fig. 1a), ADAMTS-4 protein expression levels (Fig. 1b) and ADAMTS-5 protein expression levels (Fig. 1c).

4×10^5^ degenerative cervical NP cells were cultured in collagen I scaffold for four weeks. Equivalent cell proliferation rates were determined from all samples of degeneration grade III and IV. The mean values of proliferation rates were 1.086×10^6^ (±13167) and 1.058×10^6^ (±18661) cells for grade III and IV respectively (P<0.0061). The confirmed cell proliferation rates between degeneration grade III and IV differed with about 2.6% (table 2 and figure 1a). Age and gender do not seem to play distinct role in influencing proliferation rate of degenerative cervical NP cells (data not shown).

**Table 2:** Proliferation rates of degenerative cervical NP cells in 3D culture.

DDG	Minimum	Maximum	Range %	Mean	SD	Mean difference	Mean fold
III	1067461	1105860	3,47%	1086441	13167	27656	1.0261
IV	1037469	1079638	3,9%	1058785	18661		

Fifteen specimens of degenerative cervical NP tissues with degeneration grades III and IV were acquired from 15 patients operated due to cervical disc herniation. 4×10^5^ cells from each specimen were grown for four weeks in collagen I scaffold. Cell proliferation data (MTT assay) were analyzed on the basis of disc degeneration grade (DDG). The lowest and highest values of cell numbers of the analyzed samples are presented in the columns “Minimum” and “Maximum” correspondingly. The Range is calculated as the difference between the lowest and highest values.

### Endogenous Expression Levels of Catabolic, Anti-catabolic and Inflammatory Cytokines in Degenerative Cervical NP Cell

**Figure 2:**
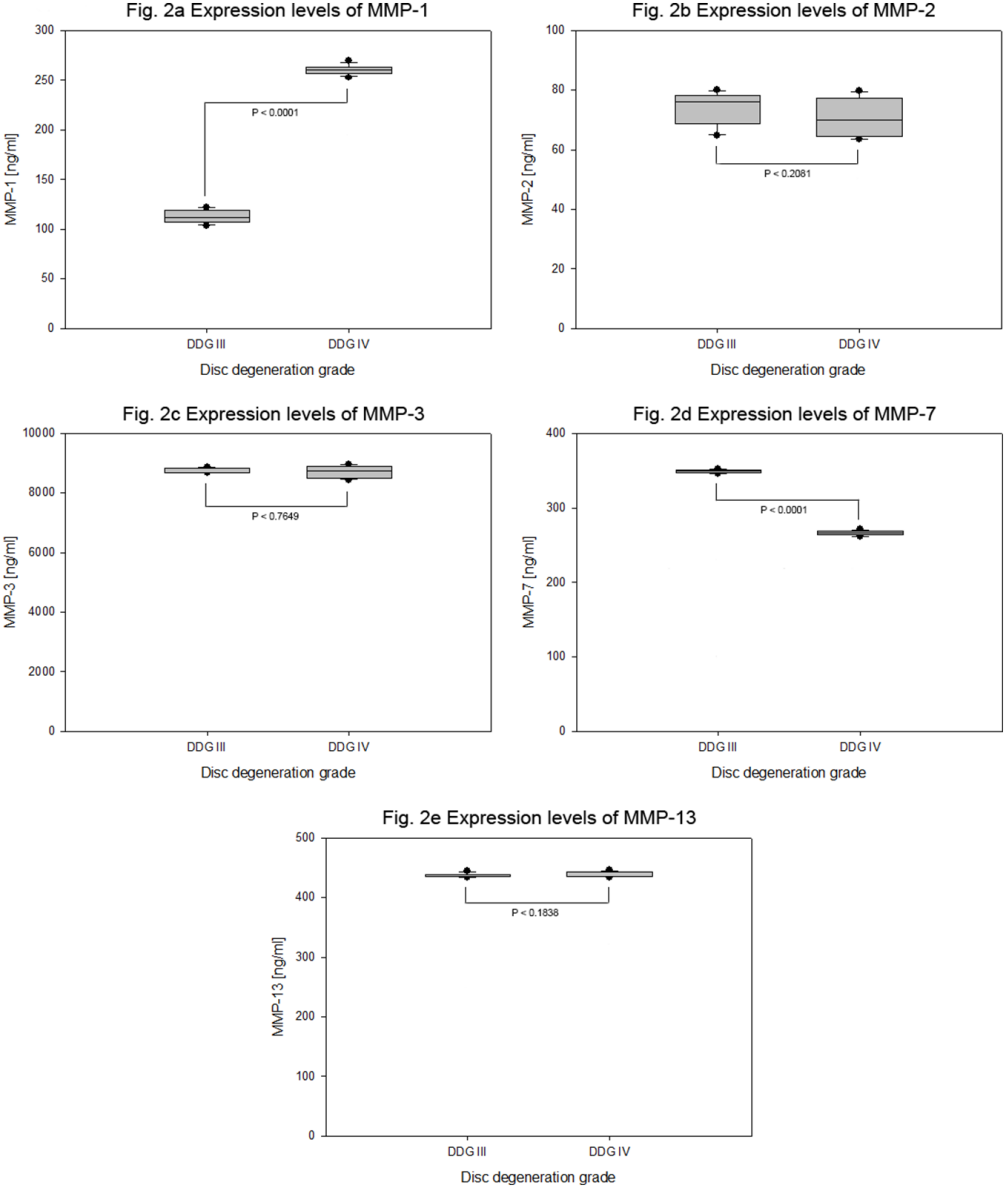
Expression levels of endogenous MMPs in degenerative cervical NP cells. Cervical NP specimens of degenerative grade III and IV were isolated from 15 patients. From each specimen 4×10^5^ cells were grown in collagen I scaffold for four weeks to determine the endogenous expression levels (ELISA) of MMPs on the basis of disc degeneration grade (DDG). For each sample 100 µg total protein extracts were applied per experiment. Whiskers min to max of box plots show MMP-1 expression levels (Fig. 2a), MMP-2 expression levels (Fig. 2b), MMP-3 expression levels (Fig. 2c), MMP-7 expression levels (Fig. 2d) and MMP-13 expression levels (Fig. 2e).

4×10^5^ degenerative cervical NP cell were cultured for four weeks in collagen I scaffold. High and increasing expression levels of the catabolic factor ADAMTS-4 with mean expression values of 1718±63.7 pg/ml and 3308±123 pg/ml were verified for degeneration grades III and IV respectively (P<0.0001), which corresponds to a 1.9 fold increase in mean expression values. Moreover, higher but equivalent expression levels of ADAMTS-5 with mean expression values 4136±191 pg/ml and 4215±160 pg/ml were confirmed for degeneration grades III and IV respectively (P<0.3979) (table 3 and figure 1b–c). MMP-3 was expressed at highest levels compared to all other catabolic factors tested. However, its expression levels in degeneration grade III and IV were equivalent with mean expression values 8749±86.8 pg/ml and 8706±211 pg/ml respectively (P<0.6251). In proportion to the expression values of MMP-3, very low but increasing expression levels of MMP-1 with mean expression values 113±7.10 pg/ml and 260±5.17 pg/ml (P<0.0001), very low and equivalent expression levels of MMP-2 with mean expression values 74±2.30 pg/ml and 70±6.63 pg/ml (P<0.3580), moderate but decreasing expression levels of MMP-7 with mean expression values of 349±2.30 pg/ml and 266±3.17 pg/ml (P<0.0001) as well as equivalent expression levels of MMP-13 with mean expression values 437±3.62 pg/ml and 440±4.45 pg/ml (P<0.1517) were determined (table 3 and figure 2a–e). MMP-8, MMP-9 and MMP-10 were expressed either at extremely low level or not expressed at all, as their expression levels remained below the minimum detectable dose (MDD) of our detection system (MDD of MMP-8<20 pg/ml, MMP-9<156 pg/ml and MMP-10<4 pg/ml).

**Table 3:** Levels of endogenous protein expression for catabolic and anti-catabolic cytokines in degenerative cervical NP cells.

Catabolic/anti- catabolic protein	DDG	Min. pg/ml	Max. pg/ml	Range %	Mean pg/ml	SD	Mean different DDG pg/ml		Mean fold
ADAMTS-4	III	1679	1860	9.730	1718	63.7	IV–III	1590	1.926
	IV	3046	3464	12.06	3308	123			
ADAMTS-5	III	3789	4287	11.61	4136	191	IV–III	79	1.019
	IV	3917	4376	10.48	4215	160			
MMP-1	III	103	121	14.88	113	7.10	IV–III	147	2.300
	IV	252	269	6.319	260	5.17			
MMP-2	III	64	80	20.00	74	5.94	IV–III	−4	0.946
	IV	63	79	20.25	70	6.63			
MMP-3	III	8672	8846	1.966	8749	86.8	IV–III	−43	0.995
	IV	8440	8961	5.814	8706	211			
MMP-7	III	345	351	1.709	349	2.30	IV–III	−83	0.762
	IV	261	270	3.333	266	3.17			
MMP-13	III	434	444	2.252	437	3.62	IV–III	3	1.006
	IV	434	445	2.471	440	4.45			
TIMP-1	III	13934	14513	3.989	14204	237	IV–III	2633	1.185
	IV	16557	17138	3.39	16837	195			
TIMP-2	III	10497	11317	7.245	10919	316	IV–III	2887	1.264
	IV	13056	14171	7.868	13806	362			
TIMP-3	III	846	897	5.685	873	21.1	IV–III	85	1.097
	IV	922	998	7.615	958	30.9			
TIMP-4	III	196	200	2.00	197	1.31	IV–III	12	1.060
	IV	199	214	7.00	209	4.45			

MMP-8, MMP-9 and MMP-10 expression levels were below the minimum detectable dose (MDD) of our detection system. MDD of MMP-8<20 pg/ml, MMP-9<156 pg/ml and MMP-10<4 pg/ml.

4×10^5^ cells from each specimen of fifteen herniated degenerative cervical NP tissues of grades III and IV were grown for four weeks in collagen I scaffold. On the basis of disc degeneration grade (DDG) the expression levels of catabolic (ADAMTs, MMPs) and anti-catabolic (TIMPs) cytokines were determined (ELISA) from 100 µg total protein extracts of each sample per experiment. In the columns “Minimum” and “Maximum” the corresponding lowest and highest values of protein expression levels (pg/ml) of the samples are presented.

**Figure 3:**
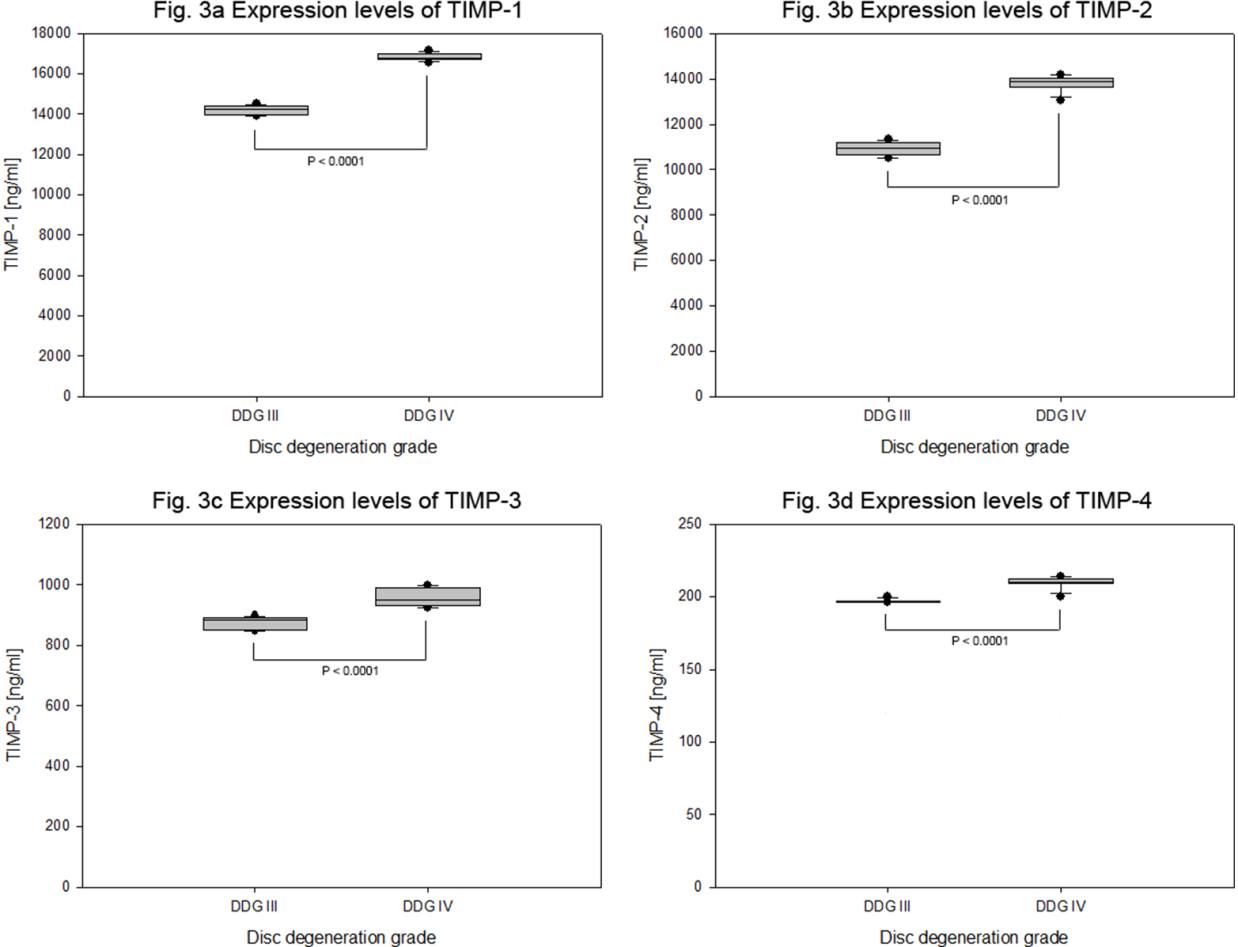
Endogenous protein levels of TIMPs in degenerative cervical NP cells. To determine the endogenous expression levels of TIMPs in cervical NP cells, 15 NP specimens of degenerative grade III and IV of herniated discs were used. In collagen I scaffold 4×10^5^ NP cells from each specimen were grown for four weeks, and on the basis of disc degeneration grade (DDG) the protein concentration of TIMPs were defined (ELISA) from 100 µg total protein extracts of each sample. TIMP-1 expression levels (Fig. 3a), TIMP-2 expression levels (Fig. 3b), TIMP-3 expression levels (Fig. 3c) and TIMP-4 expression levels (Fig. 3d) are shown by box plots with whiskers min to max.

However, higher and increasing expression levels of the anti-catabolic factors TIMP-1 and TIMP-2 countered the high expression levels of MMP-3. Their respective mean expression values were 14204±237 pg/ml and 16837±195 pg/ml for TIMP-1 (P<0.0001) and 10919±316 pg/ml and 13806±362 pg/ml for TIMP-2 (P<0.0001). Compared to the expression values of TIMP-1 and TIMP-2 the expression levels of TIMP-3 with mean expression values 873±21.1 pg/ml and 958±30.9 pg/ml (P<0.0001) were relatively very low. Among all TIMPs the lowermost expression levels were recorded for TIMP-4 with mean expression values 197±1.31 pg/ml and 209±4.45 pg/ml (P<0.0001) (table 3 and figure 3a–d).

**Figure 4:**
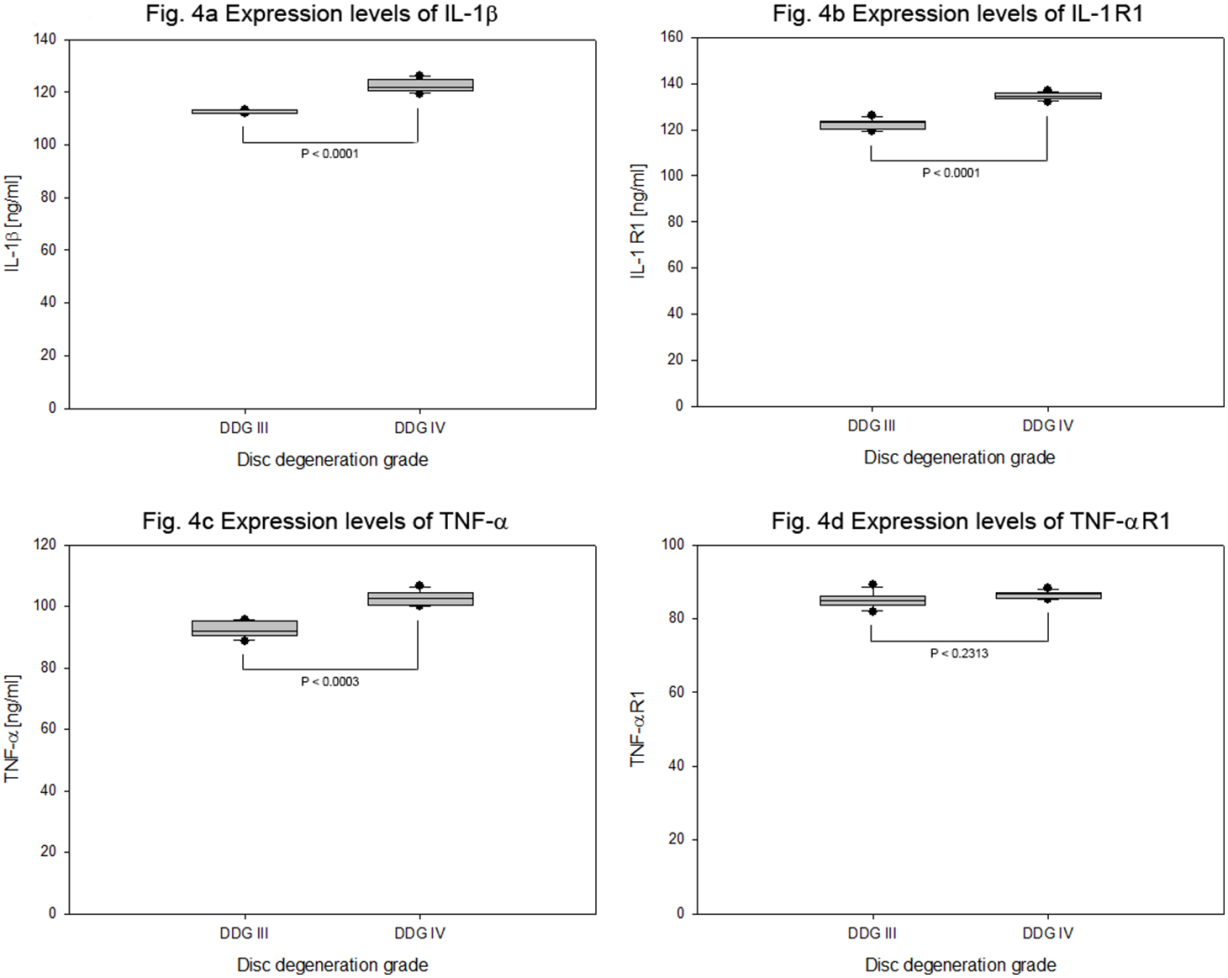
Protein expression levels of endogenous inflammatory cytokines in cervical NP cells. 15 degenerative grade III and IV herniated cervical discs were used to isolate NP specimens. Collagen I scaffold was used to culture 4×10^5^ NP cells from each specimen for four weeks. The endogenous expression levels of inflammatory cytokines were confirmed (ELISA) from 100 µg total protein extracts of each sample on the basis of disc degeneration grade (DDG). Box plots with whiskers min to max show IL-1β expression levels (Fig. 4a), IL-1 R expression levels (Fig. 4b), TNF-α expression levels (Fig. 4c) and TNF-α R1 expression levels (Fig. 4d).

The inflammatory cytokines IL-1β, IL-1 R, TNF-α and TNF-α R1 were detected relatively at very low expression levels. In degeneration grade III and IV the expression levels of IL-1β and IL-1 R were higher than that of TNF-α and TNF-α R1. Their respective mean expression values were 112±0.728 pg/ml and 122±2.55 pg/ml (P<0.0001) for IL-1β, 122±2.36 pg/ml and 134±1.61 pg/ml (P<0.0001) for IL-1 R, 92±2.80 pg/ml and 102±2.47 pg/ml (P<0.0001) for TNF-α, 85±2.26 pg/ml and 86±1.02 pg/ml (P<0.1453) for TNF-α R1. The mean expression values of IL-1β, IL-1 R and TNF-α were increased by about 10% between degeneration grade III and IV, whereas the mean expression values of TNF-α R1 remained unaffected (table 4 and figure 4a–d). The calculated significance of changes in expression levels as a function of age by decade showed slightly increasing expression levels of TNF-α (P<0.0001). Gender does not appear to play any role in influencing the expression levels of the analyzed catabolic, anti-catabolic and inflammatory cytokines (data not shown).

**Table 4:** Concentrations of endogenously expressed inflammatory cytokines, anabolic factors and matrix proteins in degenerative cervical NP cells.

Inflammatory/Matrix protein	DDG	Min. pg/ml	Max. pg/ml	Range %	Mean pg/ml	SD	Mean different DDG pg/ml		Mean fold
IL-1β	III	111	113	1.769	112	0.728	IV–III	10	1.089
	IV	119	126	5.555	122	2.55			
IL-1 R	III	119	125	4.80	122	2.36	IV–III	12	1.098
	IV	131	136	3.68	134	1.61			
TNF-α	III	88	95	7.368	92	2.80	IV–III	10	1.108
	IV	100	106	5.66	102	2.47			
TNF-α R1	III	81	89	8.988	85	2.26	IV–III	1	1.011
	IV	85	88	3.409	86	1.02			
Aggrecan	III	24590	28629	14.11	26682	1861	IV–III	−12181	0.543
	IV	13212	15971	17.28	14501	936			
Collagen II	III	9056	10345	12.46	9567	384	IV–III	−3522	0.631
	IV	5706	6315	9.643	6045	198			

Anabolic protein and Collage I expression levels were below the minimum detectable dose (MDD). MDD of BMP-2<11 pg/ml, BMP-4<1 pg/ml, BMP-6<3 pg/ml, BMP-7<2 pg/ml, IGF-1<25 pg/ml, TGF-β1, TGF-β3 and Collagen I<217 pg/ml <5 pg/ml.

For protein isolations grade III and IV cervical NP tissues of herniated discs were acquired from 15 patients and 4×10^5^ cells from each specimen were grown for four weeks in collagen I scaffold. Using ELISA the concentration of inflammatory cytokines (IL-1β, IL-1 R, TNF-α, TNF-α R), anabolic factors (BMPs, TGF-βs, IGF-1) and matrix proteins (aggrecan, collagen I and II) were determined from 100 µg total protein extracts of each sample on the basis of disc degeneration grade (DDG). The columns “Minimum” and “Maximum” show the lowest and highest values of protein expression levels (pg/ml) of the analysed samples correspondingly.

### Levels of Endogenous Anabolic and Matrix Proteins in Degenerative Cervical NP Cells

**Figure 5:**
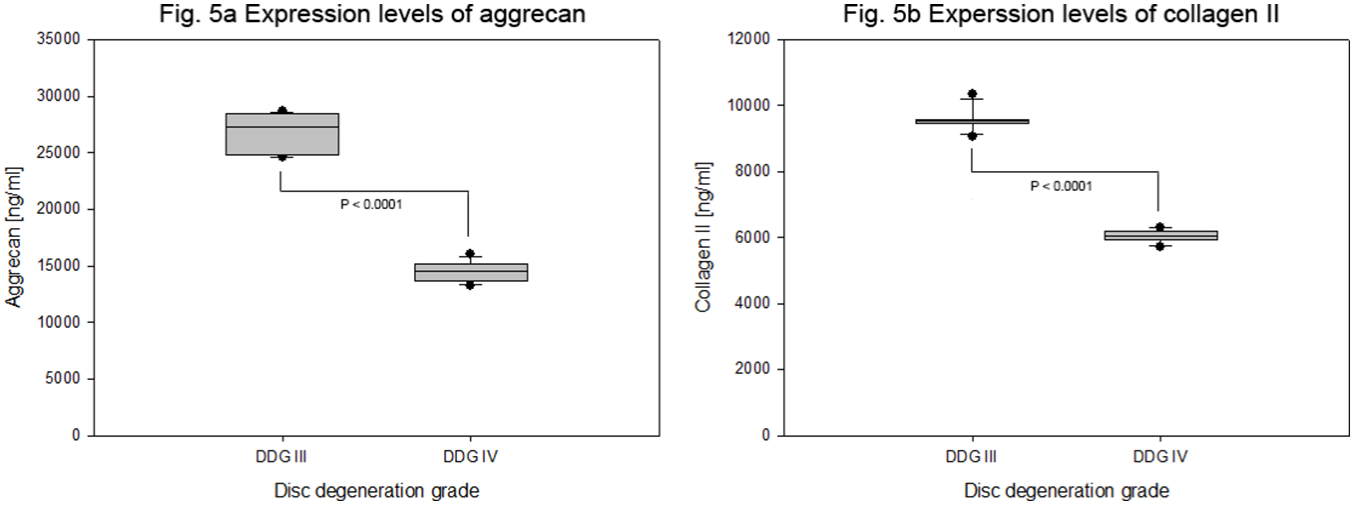
Endogenous expression levels of matrix proteins in degenerative cervical NP cells. From 15 herniated cervical discs of grade III and IV NP tissues were isolated and 4×10^5^ cells from each sample were cultured in collagen I scaffold for four weeks. On the basis of disc degeneration grade (DDG) the concentration of aggrecan, collagen I and collagen II were measured (ELISA) from 100 µg total protein extracts of each sample. Aggrecan expression levels (Fig. 5a) and collagen II expression levels (Fig. 5b) are shown using box plots with whiskers min to max. Collagen I expression level remained below minimum detectable dose of our detection system (table 4).

Decreasing expression levels of matrix proteins aggrecan and collagen II were determined in degenerative cervical NP cells of degeneration grade III and IV. The respective mean expression values of aggrecan were 26682±1861 pg/ml and 14501±936 pg/ml (P<0.0001) and of collagen II were 9567±384 pg/ml and 6045±198 pg/ml (P<0.0001). The mean expression values of aggrecan and collage II in degeneration grade III were about 1.8 and 1.6 fold higher than that in degeneration grade IV (table 4 and figure 5a–b). The expression level of collagen I in cervical NP cells remained below the minimum detectable dose (MDD) of our detection system (MDD<217 pg/ml). Similarly in control 3D culture (agarose gel) and control 2D culture (TC dishes) the expression level of collagen I in cervical NP cells remained below the minimum detectable dose (data not shown). Moreover, the expression levels of the analyzed anabolic factors remained below the minimum detectable dose (MDD) of our detection system (MDD<1–25 pg/ml). They were expressed either at extremely low level or not expressed at all. The calculated significance of changes in expression levels as a function of age by decade showed decreasing expression levels of aggrecan (P<0.0001) and collagen II (P<0.0001). Gender does not appear to play any role in influencing the expression levels of the anabolic and matrix proteins (data not shown).

## Discussion

In previous publications the issues of biological treatment approaches in degenerative cervical disc diseases have not been reported, even though cervical disc degeneration, which could be associated with pain and loss of mobility, is clinically important. So far, the pathology of cervical disc degeneration has been studied only from a morphologic view point using MRI [1]–[6]. Different biological treatment approaches, which were carried out in degenerative lumbar discs, have shown the importance of NP cells for the maintenance of IVD matrix. They coordinate the expression of anabolic, catabolic, anti-catabolic and inflammatory cytokines that affect the synthesis or degradation the IVD matrix.

IVD degeneration is associated with imbalances of these factors combined with the declined cell density in adult IVDs [11]–[23]. Therefore, accurate knowledge regarding the quantity and quality of these factors is vital for designing of rational gene therapeutic approaches. So far proliferation rates of degenerative cervical NP cells and their levels of endogenously expressed therapeutic targets have not yet been investigated.

Therefore, we analyzed the proliferation rates of degenerative cervical NP cells and their endogenous protein expression levels of 28 anabolic, catabolic, anti-catabolic and inflammatory cytokines as well as matrix proteins. Cervical IVD specimens of degeneration grade III and IV from 15 adult patients, operated due to cervical disc herniation, were used.

Inclusion criteria for surgery were radiographically determined intervertebral disc herniation with nerve root compression on MRI, which correlated to primary symptoms that remained unresponsive to non-operative treatment for six weeks or demonstrated progressive neurological deterioration in the face of conservative treatment. Our results may therefore be representative of degenerative cervical discs from such patients.

We determined comparable proliferation rates of cervical NP cells between degeneration grades III and IV (table 2, figure 1a). Age and gender do not seem to play a role in influencing proliferation rates of degenerative cervical NP cells. The data might suggest that progressive degenerative changes in adult IVDs may not be triggered by decreasing of NP cells. This might occur instead because of undesirable phenotypic changes in NP cells. Previous studies of lumbar NP cells have also indicated similar cell densities in adult age of different disc degeneration grades [24], [28]. Conversely, some studies of lumbar NP cells reported a decline of NP cell densities with increasing degeneration grades [29]–[31]. Certainly, during adolescence IVDs have considerably reduced cell density than during childhood, for the reason that the cell environment within the disc severely changes during growth. During growth IVD size increases and blood supply as well as diffusion decrease, which result in cell death triggered by decreased concentration of glucose and oxygen [32]–[37].

High and increasing expression levels of catabolic factor ADAMTS-4 with about 1.9 fold upturn were recorded between degeneration grades III and IV (table 3, figure 1b–c). Additionally, high but comparable expression levels of ADAMTS-5 were recorded in both grades of degeneration. In contrast to the previous finding, which showed the age of patients is associated with the percentage of immunohistological ADAMTS-5 positive lumbar disc cells [38], we found here age and gender to be independent factors regarding the expression ADAMTS-4 and ADAMTS-5. These data, supporting the previous report on lumbar NP cells [24], indicate that ADAMTS-4 and ADAMTS-5 could represent attractive targets for biological treatment approaches of degenerative disc diseases. Moreover, they have been shown to cause aggrecan degradation in human osteoarthritic cartilage [39]–[41].

Compared to all other tested catabolic factors MMP-3 was expressed at highest levels, but its expression levels did not alter with increasing grades of degeneration. Its mean expression level was about 33 fold of MMP-1, 118 fold of MMP-2, 25 fold of MMP-7 and 20 fold of MMP-13 (Table 3 and figure 2a–e). MMP-8, MMP-9 and MMP-10 were expressed either at extremely low level or not expressed at all, as their expression levels remained below the minimum detectable dose (table 3).

In contrast to a previous report, where immunohistological staining was negative for TIMP-1 in MMP-3 positive stained surgical lumbar specimens [42], the high expression of MMP-3 in degenerative cervical NP cells was counteracted by even higher and increasing expression levels of TIMP-1 (1.9 fold of MMP-3) and TIMP-2 (1.6 fold of MMP-3). In comparison significantly less TIMP-3 and even far less TIMP-4 expression levels were recorded. Their respective mean expression values were about only 11% and 1.2% of TIMP-1 (table 3 and figure 3a–d). The expressions of MMPs and their counterparts TIMPs in lumbar NP cells have been controversy discussed. Consistent and substantial up-regulated mRNA levels of MMP-3 and MMP-8 were observed and these up-regulations were paralleled by greater expression of TIMP-1 and not TIMP-2 [20]. Moreover the most extensive immunohistochemical stainings were seen for MMP-1, MMP-2, MMP-3, and MMP-9 and much less for MMP-7 and MMP-8, and these up-regulations were paralleled by greater expression of TIMP-2 and not TIMP-1 [41]. Furthermore the number of immunopositive cells for MMP-1, MMP-3, MMP-13 and ADAMTS-4 increased with the severity of degeneration and this was accompanied by increased number of immunopositive cells for TIMP-1 and TIMP-2 but not for TIMP-3 [19]. Our data suggest that ectopic expression of TIMP-3, an inhibitor of ADAMTs, and repression of MMP-3 would be more interesting to improve the regeneration potential of degenerative cervical NP cells. On the other hand, as TIMP-1 and TIMP-2, inhibitors of MMP-3, are expressed at higher levels than MMP-3, their ectopic expression might not be potentially effective. It would be quite more interesting to focus on their mutational and posttranslational alterations.

The inflammatory cytokines IL-1β and TNF-α as well as their receptors IL-1 R and TNF-α R1 were expressed relatively at very low levels. However, higher expression levels of IL-1β and IL-1 R were detected in degeneration grade III and IV than that of TNF-α and TNF-α R1. Moreover, increased mean expression values of about 10% were recorded between degeneration grade III and IV for IL-1β, IL-1 R and TNF-α, whereas the mean expression values of TNF-α R1 remained unaffected (table 4 and figure 4a–d). Based on our data, IL-1β, IL-1R as well as TNF-α might be involved in the pathogenesis of cervical disc degeneration, where IL-1β and IL-1 R might act as better therapeutic targets. Furthermore, treatment of lumbar disc cells with 10 ng/ml recombinant IL-1β has shown increased expression levels of ADAMTS-4, MMP-3 and MMP-13 with decreased expression levels of aggrecan, collagen II, collagen I and SOX6 [43]. Additionally, real time PCR and immunohistochemistry studies have previously shown the expression of IL-1β, IL-1 R, TNF-α and TNF-α R1 in lumbar NP cells of healthy and degenerative discs, and their expression levels increased with increasing degeneration grades [21], [41].

We determined with increasing grades of degeneration decreased expression levels of matrix proteins aggrecan and collagen II in cervical NP cells. Between degeneration grade III and IV about 1.8 and 1.6 folds of decreased aggrecan and collagen II expressions were recorded respectively (table 4 and figure 5a–b). The expression level of collagen I remained below the minimum detectable dose of our detection system (table 4). Moreover, the expression level of collagen I in cervical NP cells remained also below the minimum detectable dose (data not shown) in control 3D culture (agarose gel) as well as in control 2D culture (TC dishes). The calculated significance of concentration changes as a function of age by decade showed decreasing expression levels of aggrecan (P<0.0001) and collagen II (P<0.0001). Gender does not appear to play any role in influencing the expression levels of matrix proteins (data not shown). Degeneration grade and age correlated changes were also shown in lumbar disc cells [24], [44]–[45].

Growth factors have been shown to be important biological components to stimulate matrix synthesis [11], [46]. However, their endogenous expression levels in cervical IVD cells have never been studied. Using immunohistochemical analysis only a few number of publications have presented the expression of growth factors bFGF, TGF-β1, TGF-β2 and growth factor receptors TGFβ RII, FGF R3 and BMP RI in lumbar IVDs [47], [22]. Although we applied a large amount of total protein extract from cervical NP cells (100 µg), the protein expression levels of all tested growth factors remained in contrast below the minimum detectable doses. In addition, the minimum detectable doses of the tested growth factors are very low (table 4). Hence, our data emphasize imbalances between the expression levels of anabolic and catabolic proteins in degenerative cervical NP cells, which might result in a catabolic inflammatory metabolism of the disc matrix.

Therefore, the endogenous protein expression data of the anabolic, catabolic, anti-catabolic and inflammatory cytokines in degenerative cervical NP cells suggest that suppression of the catabolic factors (MMP-3, ADAMTS-4 and ADAMTS-5) along with the inflammatory cytokines (IL-1β and IL-1 R) might be a favorable gene therapeutic approach. This approach could be combined with ectopic expression of the anabolic factors and the anti-catabolic factor TIMP-3, an inhibitor of ADAMTS-4 and ADAMTS-5. The joint course might improve the regeneration potential of degenerative cervical disc cells.
