# Correction: Imbalanced Protein Expression Patterns of Anabolic, Catabolic, Anti-Catabolic and Inflammatory Cytokines in Degenerative Cervical Disc Cells: New Indications for Gene Therapeutic Treatments of Cervical Disc Diseases

**DOI:** 10.1371/journal.pone.0107091

**Published:** 2014-08-25

**Authors:** The PLOS ONE Staff

## Notice of Republication

This article was republished on August 7, 2014, to replace incorrectly changed characters in the byline, citation, and references. The publisher apologizes for the errors. Please download this article again to view the correct version. The originally published, uncorrected article and the republished, corrected article are provided here for reference.

## Supporting Information

File S1 Originally published, uncorrected article.
(PDF)

File S2 Republished, corrected article.
(PDF)
